# Selection of Non-*Saccharomyces* Yeasts from Extreme Oenological Environments for Potential Use in Winemaking

**DOI:** 10.3390/microorganisms13061260

**Published:** 2025-05-29

**Authors:** María Trinidad Alcalá-Jiménez, Juan Carlos García-García, Juan Carlos Mauricio, Juan Moreno, Rafael Peinado, Teresa García-Martínez

**Affiliations:** Department of Agricultural Chemistry, Edaphology and Microbiology, Agrifood Campus of International, Excellence CeiA3, Universidad de Córdoba, 14014 Córdoba, Spain; b52aljim@uco.es (M.T.A.-J.); p22gagaj@uco.es (J.C.G.-G.); qe1movij@uco.es (J.M.); qe1peamr@uco.es (R.P.); mi2gamam@uco.es (T.G.-M.)

**Keywords:** *Zygosaccharomyces bailii*, *Hanseniaspora opuntiae*, *β*-glucosidase activity, killer phenotype, aroma enhancement

## Abstract

This study evaluated the oenological potential of two non-*Saccharomyces* yeast strains, *Hanseniaspora opuntiae* TR-5 and *Zygosaccharomyces bailii* L-25, isolated from extreme winemaking environments in southern Spain. Out of 156 yeast isolates screened from high-sugar musts and flor yeast biofilms, strains were selected based on their *β*-glucosidase activity, killer phenotype, and ethanol production, traits associated with aroma release and microbial competition. Fermentation trials on sugar-rich synthetic medium showed that both *H. opuntiae* and *Z. bailii* achieved ethanol yields of 10% *v*/*v* and residual sugars at 4 g/L. Co-culture and sequential inoculation, with *H. opuntiae* introduced first and *Z. bailii* added on day four, resulted in complete alcoholic fermentation and a reduction in undesirable acetoin levels compared to single-strain fermentations. These findings highlight the practical potential of using selected non-*Saccharomyces* strains in sequential fermentations to improve aroma complexity, fermentation reliability, and sensory quality in wines, even in the absence of *Saccharomyces cerevisiae*. The application of these strains offers a novel approach for precision oenology and varietal expression in challenging musts.

## 1. Introduction

The variability of the microbiota involved in wine fermentation can influence the stability and quality of the wine [1]. Yeasts have an important role during the fermentation of the must by carrying out alcoholic fermentation. However, not all species that make up the wine microbiome contribute pleasant aromas, and this poses a challenge for oenology. The participation of non-*Saccharomyces* yeasts in winemaking can produce a complexity of compounds, including esters, alcohols, and acids, compared to pure *Saccharomyces* fermentations [2,3]. Therefore, the selection of yeast strains used in the fermentation process is crucial to improve the fermentation conditions. This is why the need for precision in yeast selection is so important. In fact, non-*Saccharomyces* yeasts have traditionally been regarded as undesirable in winemaking due to their limited fermentative capacity and association with spoilage, with *Brettanomyces* and *Pichia* serving as classic examples. *Brettanomyces* is notorious for producing off-flavors such as barnyard, medicinal, or phenolic notes, which can compromise wine quality even at low concentrations. Similarly, certain *Pichia* species are linked to the development of surface films and the production of volatile phenols and ethyl acetate, leading to sensory defects and diminished wine stability [4]. However, the role of non-*Saccharomyces* yeasts in oenology is increasingly recognized as dual and context-dependent. While some species and strains can cause spoilage, others contribute positively to fermentation and wine complexity. Recent research has demonstrated that selected non-*Saccharomyces* yeasts can enhance the aromatic profile, mouthfeel, and microbial stability of wines. For example, genera such as *Hanseniaspora*, *Lachancea*, and *Zygosaccharomyces* have been shown to release varietal aroma compounds, produce desirable esters, and even inhibit spoilage organisms through killer activity or competition for nutrients. However, there are now many studies that have shown that non-conventional yeasts have positive effects during winemaking from different grape musts. Furthermore, wine obtained from spontaneous fermentation is superior to simple *Saccharomyces cerevisiae* fermentation in complexity, body, aroma, and terroir characteristics [5]. Aroma plays a fundamental role in wine selection and consumer acceptance. The aromatic profile or volatilome of wine is composed of several odorants, influencing its flavor [6].

Studies have confirmed that the genus *Hanseniaspora* can positively influence the color, flavor, aroma, and stability of wines [7,8,9]. Within this genus, *Hanseniaspora uvarum* is one of the most predominant species, which is one of the most predominant non-*Saccharomyces* yeast species found in grapes [10]. This species contributes to the natural aroma of wine when used in mixed fermentations. However, if this yeast is not present during fermentation, the resulting wine will lack aromatic complexity, as observed in the study conducted by Rementeria et al. [11]. On the other hand, the *Hanseniaspora opuntiae* species has a fermentative power of less than 6% *v*/*v*; however, it has been proposed that this yeast be used in mixed fermentations with *Lachancea thermotolerans,* as it may improve the freshness and fruitiness of wines [12].

*Zygosaccharomyces bailii* is recognized as an osmotolerant yeast with a high fermentative power. This yeast strain can grow under conditions of ≥18% *v*/*v* ethanol. The study carried out by Santos et al. [13] showed that this strain was able to metabolize 25 g/L of fructose, a characteristic that would be advantageous to restart or terminate fermentation [14]. Some selected strains of *Zygosaccharomyces* spp. could be useful because they can produce high levels of ethyl esters [15], such as in the study carried out by Garavaglia et al. [16] in Chardonnay wine. Furthermore, these strains can produce high levels of 2-phenylethyl acetate, isoamyl acetate, ethyl acetate, and ethyl esters, the latter being very important, since they contribute fruity and floral aromatic notes to the wine [16]. Solieri [17] describes this species as promising to produce lactic acid and ethanol. Furthermore, *Z. bailii* strains that have killer activity produce a toxin known as “zygocin”, a toxin encoded by dsRNA. This study aimed to characterize the oenological potential of *H. opuntiae* and *Z. bailii* yeast strains, evaluating their contributions to improving wine quality during fermentation processes. By analyzing their metabolic profiles and fermentative behavior, the research aimed to discover how these yeast strains could improve key oenological attributes such as aroma complexity, microbial stability, and overall sensory characteristics of wines. Our initial hypothesis is that strains adapted to extreme environments (high ethanol and sugar content) may exhibit greater fermentation and a better contribution to aroma. To this end, strains from extremophile environments were isolated and characterized. From the strains selected based on the enzyme parameters analyzed, the fermentative potential of different non-*Saccharomyces* yeasts present in hostile natural environments was evaluated. These include very sugary musts from the Montilla-Moriles Protected Designation of Origin (PDO), a warm-climate region, characterized by high sugar concentrations and where the predominant non-*Saccharomyces* yeast species are in a sugar-rich environment, and therefore adapted to these, allowing them to survive the initial concentrations of the medium used for this oenological characterization. Yeasts from the biologically aged biofilms/veils wines from the Jerez–Xérès–Sherry PDO, characterized by high alcohol concentrations, where the predominant species can complete the fermentation of these sugars, and, therefore, complete the fermentation. The Montilla-Moriles and Sherry PDOs were chosen for this study because they represent extreme and contrasting oenological environments—Montilla-Moriles is characterized by musts with high sugar concentrations, while Jerez–Xérès is known for biologically aged wines with high ethanol content and unique flor yeast biofilms—offering exceptional microbial biodiversity and presenting distinct fermentation challenges that are ideal for isolating and evaluating robust, adaptive non-*Saccharomyces* yeast strains.

To our knowledge, this is one of the few studies evaluating *β*-glucosidase and killing the activity of wild yeasts from both extremophile oenological environments, based on two PDOs in Andalusia (Spain), in addition to performing a fermentation analysis of pure and mixed culture strains of the selected species: *H. opuntiae* TR-5 and *Z. baili* L-25.

The duality underscores the importance of strain selection and controlled application. Rather than being dismissed solely as spoilage agents, non-*Saccharomyces* yeasts should be evaluated for both their risks and their potential technological benefits in precision oenology. The present study builds on this perspective by exploring the oenological potential of *H. opuntiae* and *Z. bailii*, two non-*Saccharomyces* species with promising enzymatic activities and fermentative traits, aiming to harness their positive contributions while managing possible drawbacks.

## 2. Materials and Methods

### 2.1. Isolation of Autochthonous Wine Yeasts

Wine yeasts were isolated from the two aforementioned wine-growing regions in southern Spain. These yeasts were harvested and isolated using aseptic techniques from natural musts from the Montilla-Moriles PDO region in Córdoba, Spain. In this study, yeasts were isolated during the spontaneous fermentation of these musts with more than 250 g/L sugars, using serial dilutions and different screening methods in the laboratory. The natural must is made from the Pedro Ximénez grape variety. However, the yeasts isolated from the Jerez–Xérès–Sherry PDO (Cádiz, Spain) were harvested from flor yeast biofilms during the biological ageing of Fino wine using a sterile spatula. For this study, the yeasts were isolated using serial dilution and enrichment methods. Sherry PDO Fino wine is made from a different grape variety known as Palomino. The must and the flor yeast were independent sample types.

A total of 156 isolates were obtained from the two areas studied. During the spontaneous fermentation of must from vineyards in the Montilla-Moriles area, a total of 85 yeasts were obtained from the Coop La Union (Montilla, Córdoba, Spain) and the Coop Vitivinícola Aguilar de la Fra (Aguilar de la Frontera, Córdoba, Spain). A total of 71 isolates were obtained from two of the most prominent sherry producers in Jerez: the Williams & Humbert (Jerez, Cádiz, Spain) and Gonzalez Byass wineries (Jerez, Cádiz, Spain). The selection of yeast species from these environments was aimed at comparing strains from opposing difficult environments. The strains isolated from Córdoba came from musts with a high sugar concentration, while the strains from Cadiz came from an environment with a high ethanol concentration (over 15.5% *v*/*v*), no fermentable sugars, and a low nitrogen source.

### 2.2. Culture Media and Enzymatic Screening Procedures

#### 2.2.1. WL Nutrient Agar (Wallerstein Laboratory Medium)

According to the manufacturer’s instructions (Oxoid, Basingstoke, UK), WL and a specific medium were prepared from its dehydrated formulation by dissolving 6% (*w*/*v*) of the preparation in distilled water, together with 2% (*w*/*v*) of agar. The mixture was autoclaved at 120 °C for 15 min. After being poured onto Petri plates, these were incubated at 28 °C for a period of three days. The resulting medium allowed effective distinction of the different yeast genera, thanks to the colony shape and colorimetric variations observed, thus providing a consistent visual tool for their identification [18]. The WL medium is a selective medium, and thanks to the green-bromocresol indicator, the yeasts, depending on their acidification, acquired a different colorimetry ranging from white to different shades of green, allowing different yeast strains to be selected.

#### 2.2.2. Lysine Medium

For the confirmation of isolates belonging to non-*Saccharomyces* yeasts, a selective medium prepared according to the procedure described by Alcalá-Jiménez et al. [18] was used. Positive identification was established when microbial growth was observed after two successive subcultures, which was considered a potential verification criterion.

#### 2.2.3. Determination of Yeast Killer Activity

Killer activity was measured using the method described by Ramírez et al. [19]. Yeast was analyzed on methylene blue plates at pH 4.0 according to the method described by Kaiser et al. [20]. Plates were seeded with 100 μL of a culture of the sensitive strain previously grown for 48 h. The strains to be studied for their killer activity were patched from solid cultures and seeded in triplicate on the seeded MB plates. Subsequently, the plates were incubated for 7 days at 21 °C.

#### 2.2.4. Medium for Detecting β-Glucosidase Activity

The enzymatic activity of *β*-glucosidase was measured qualitatively. It was evaluated as a medium containing 0.5% (*w*/*v*) arbutin (Sigma–Aldrich Chemie GmbH, Taufkirchen, Germany), 0.1% (*w*/*v*) yeast extract (Oxoid, Basingstoke, United Kingdom), 1% (*v*/*v*) ferric chloride solution, and 2% (*w*/*v*) agar (PanReac Castellar del Vallès, Barcelona, Spain) [21]. This medium was autoclaved at 120 °C for 15 min. The cultures were incubated at 28 °C for 14 days. This medium is based on the hydrolysis of arbutin through the action of *β*-glucosidase activity.

#### 2.2.5. YPD Agar Medium

Yeast extract peptone dextrose (YPD) agar medium is composed of 1% (*w*/*v*) yeast extract (Oxoid, Basingstoke, UK), 2% (*w*/*v*) peptone (Oxoid, Basingstoke, UK), 2% (*w*/*v*) glucose (Oxoid, Basingstoke, United Kingdom), and 2% (PanReac Castellar del Vallès, Barcelona, Spain) (*w*/*v*) agar. It is the most widely used medium for developing and maintaining yeast growth, since it is rich in amino acids, nucleotide precursors, vitamins, and essential metabolites necessary for optimal cell growth.

In the alcoholic fermentation experiments, a modified version of the YPD medium was employed, but features a notable adjustment: it incorporates a high sugar concentration of 20%, equally divided between glucose and fructose, to simulate the high sugar concentration of must.

### 2.3. Yeast Identification

Yeast identification was carried out using Matrix-Assisted Laser Desorption/Ionization Time-of-Flight Mass Spectrometry (MALDI TOF) (Bruker Daltonics, ultraflextreme, Bremen, Germany) at the Central Research Support Service (SCAI) of the University of Córdoba (UCO).

In the process of preparing the samples for analysis using MALDI-TOF-MS, the yeast was grown in the YPD agar medium used for their short-term preservation, and was dispensed into Eppendorf (Hamburg, Germany) vials, in which 300 μL of Milli-Q water and 900 μL of absolute ethanol (Merck, Darmstadt, Germany) were added.

### 2.4. Alcoholic Fermentation Tests

As the final phase of the oenological characterization process for wine yeasts, an alcoholic fermentation test was included, the objective of which was to determine their fermentative power. The basis for the selection of these non-*Saccharomyces* yeasts is their potential to ferment, modulate, and improve the flavor and aroma of wine, due to the presence of *β*-glucosidase activity. Furthermore, these yeasts could also be used as biological control yeasts, as they exhibit a killer phenotype.

In this study, fermentation tests were conducted with strains of different non-*Saccharomyces* yeast species, specifically *Hanseniaspora opuntiae* and *Zygosaccharomyces bailii*.

The conditions used here were monoculture to determine how they performed alone in a medium with optimal conditions; in co-culture, to determine how they behaved together; and, finally, in sequence, to determine if there was a reduction in ethanol content as in the study carried out by Urtubia et al. [22].

#### 2.4.1. Preparation of Pre-Inocula

The yeast strains were seeded in 100 mL flasks containing liquid YPD. These flasks underwent shaking for two days at 100 rpm to promote the growth of the yeasts selected for the alcoholic fermentation trials. The total number of cells present in the pre-inocula was then calculated using the Neubauer chamber (Paul Marienfeld GmbH&Co.KG, Lauda-Königshofen, Germany) to establish the inoculation volume necessary to correctly inoculate the different yeast strains in the different cultures during the alcoholic fermentation tests.

#### 2.4.2. Fermentation Monitoring 

Fermentations were carried out in 250 mL flasks with 150 mL of modified YPD medium; each flask was inoculated with a total of 2 × 10^6^ cells/mL. Three different conditions were used in the fermentation tests, with three biological replicates of each. The first condition was in monoculture of both selected strains, where the fermentative efficiency of each of these strains was tested. The second condition was in co-culture (CO), inoculated together at the beginning of the fermentation, in the same proportion (1:1), and in sequential culture (SQ). In the latter case, *H. opuntiae* was inoculated first, since this genus of yeast is the first to appear during spontaneous fermentation, and on the fourth day, the *Z. bailii* strain was inoculated. After inoculation, the flasks were kept in the culture chamber at 21 °C and under constant and slight agitation. Fermentation kinetics were monitored via the weight loss of the different flasks, following the method described by Spedding [23]. Fermentation was considered complete when no weight changes were observed for a couple of days.

### 2.5. Determination of Microbiological Parameters

The total number of cells present was calculated in the corresponding dilution using the Neubauer chamber (Paul Marienfeld GmbH&Co.KG, Lauda-Königshofen, Germany). To monitor the yeast population present in the different cultures daily, the number of viable cells was analyzed. To perform the study, one mL was first removed from each replicate, and serial dilutions were made. These cells were plated on WL plates to perform viable cell counts. This count was performed after 48 h of incubation at 28 °C.

### 2.6. Determination of Oenological Parameters

Oenological parameters such as pH, titratable acidity, volatile acidity, and residual sugar content were determined according to the Organisation Internationale de la Vigne et du Vin (OIV), following official European Economic Community (EEC) methods [24]. Ethanol was measured via dichromate oxidation [25].

### 2.7. Analytical Determinations of Major Aroma Compounds

Major volatile compounds were quantified with a gas chromatograph HP 6890 Series II equipped with a capillary column with molten silica CP-WAX 57 CB (50 m in length, 0.25 mm in internal diameter, and 0.4 μm in coating thickness) and a Flame ionization detector. Chromatographic conditions and sample preparation were those described by Peinado et al. [26]. The identification and quantification of the major volatile compounds were carried out by using standards submitted to the same treatment as the analyzed samples.

### 2.8. Statistical Analysis

To identify differences between the different strains used, the parameters and aromatic compounds analyzed were subjected to statistical analysis using the Statgraphics Centurion statistical package (v. 16.1.11). All experiments were performed in triplicate. All quantified compounds were subjected to simple ANOVA, and P tests were used to establish homogeneous groups (different letters indicate significant differences at a 95% confidence level); moreover, principal component analysis was conducted.

## 3. Results and Discussion

### 3.1. Microbiological Analysis

**Figure 1:**
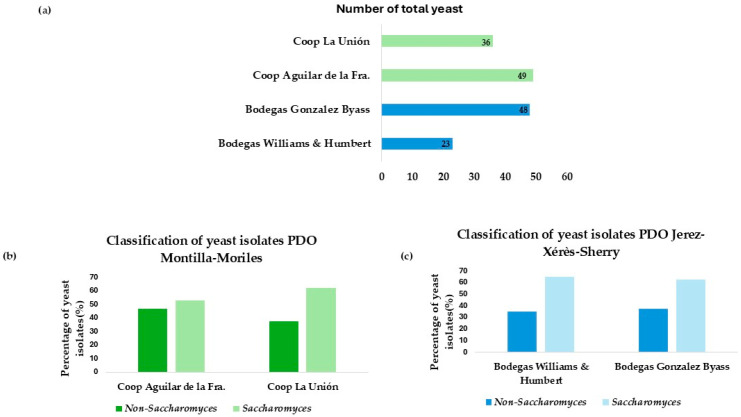
(**a**) Total number of isolates differentiated by wineries and cooperatives. (**b**) Number of isolates from each winery, differentiating between the percentage of *Saccharomyces* and non-*Saccharomyces.* (**c**) Number of isolates from each cooperative, differentiating between the percentage of *Saccharomyces* and non-*Saccharomyces*.

Based on the appearance of the colony in WL and lysine medium, a total of 156 yeasts were isolated from the two sources. These isolates are distributed as shown in Figure 1a.

A percentage differentiation was made between *Saccharomyces* and non-*Saccharomyces* among the different winemaking environments. A higher number of isolates corresponding to the non-*Saccharomyces* genus was observed in vineyards in the Montilla-Moriles area, as shown in Figure 1b. However, Figure 1c, corresponding to the flor veil isolates from the wineries of Jerez de la Fra., shows that the highest percentage of isolates obtained belong to the *Saccharomyces* genus. This is because non-*Saccharomyces* yeasts are those that appear first during spontaneous fermentation, as shown in the study by Miranda et al. [27]. Therefore, there is a higher percentage of this type of yeast in the Montilla-Moriles area, as they are isolated from fermented musts. The isolations obtained in Jerez belong to biologically aged yeasts, where the predominant species is *Saccharomyces cerevisiae*, as reported in the study by Ruiz-Muñoz et al. [28].

**Figure 2:**
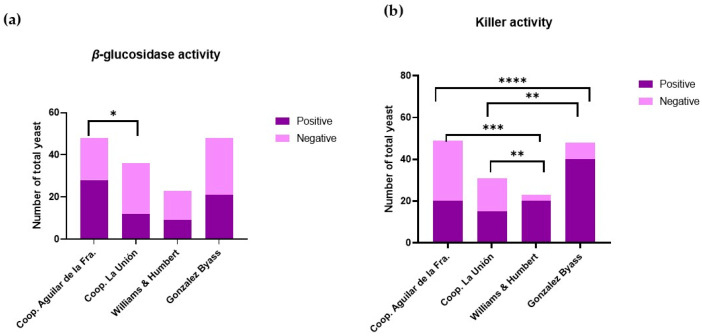
(**a**) Absence and presence of *β*-glucosidase activity in Montilla-Moriles yeast (Córdoba, Spain) and POD Jerez–Xérès–Sherry yeast (Cádiz, Spain). (**b**) Absence and presence of the killer phenotype in Montilla-Moriles yeast (Córdoba, Spain) and POD Jerez–Xérès–Sherry yeast (Cádiz, Spain). Bar plot with significant differences: * means q < 0.05; ** means q < 0.01; *** means q < 0.001; and **** means q < 0.0001.

Figure 2a shows the qualitative activity of the *β*-glucosidase enzyme observed in different viticultural environments. In grapes, some of the aromatic compounds are present as non-volatile glycosidic precursors [29]. *β*-glucosidase is an enzyme involved in the hydrolysis of substrates with glycosidic bonds, thus releasing glucose and aglycones. This enzyme is responsible for the cleavage of glycosidic bonds, releasing free terpenes, phenylpropenes, and aliphatic esters into the medium [30]. Because of this catalytic activity carried out by *β*-glucosidase, these glycosides release volatile aromatic compounds, improving the organoleptic properties [31]. This contributes to the development of the varietal aroma and the typicality of wine [32]. Consequently, as can be seen in Figure 2a, it can be concluded that yeasts from both wine-growing areas have a high potential to improve the aromas associated with compounds derived from glycosides.

Figure 2b shows that yeasts found in different wine-growing environments exhibit a killer phenotype, implying the presence of natural biological control. This activity is associated with changes in population patterns, which influence wine fermentation, as suggested by Englezos et al. [33]. Killer activity is carried out by yeasts that produce “killer substances”. These substances are of protein origin and have an antimicrobial function, inhibiting the growth and development of yeast strains susceptible to these toxins, but not their own producers [34].

### 3.2. Yeast Identification and Selection

Non-*Saccharomyces* yeast species found and identified in this study were *Hanseniaspora opuntiae* (four strains), *Lachancea thermotolerans* (four strains), *Rhodotorula mucilaginosa* (one strain), *Torulaspora delbrueckii* (three strains), *Kluyveromyces marxianus* (one strain), *Hanseniaspora uvarum* (one strain), *Pichia kudriavzevii* (two strains), and *Pichia terricola* (two strains) in Montilla-Moriles musts and, in Jerez veils, *Zygosaccharomyces bailii* (two strains) and *Torulaspora delbrueckii* (five strains). These strains were selected over the rest of the identified species because they presented both microbial properties described in Section 2. For this study, strains were selected that exhibited positive killer and *β*-glucosidase activity and produced high levels of ethanol in a synthetic medium at concentrations greater than 10% *v*/*v*. The selected strains were *H. opuntiae* TR-5 from the Montilla-Moriles Protected Designation of Origin (PDO) and *Z. bailii* L-25 from the Sherry PDO.

It should be noted that non-*Saccharomyces* strains have great potential in winemaking and should not be discarded prematurely. As some characteristics are strain-specific, isolates should not be discarded at the initial stage of research. While some strains can be selected for further studies, the potential value of others can be recognized for future research.

### 3.3. Fermentation Kinetics

**Figure 3:**
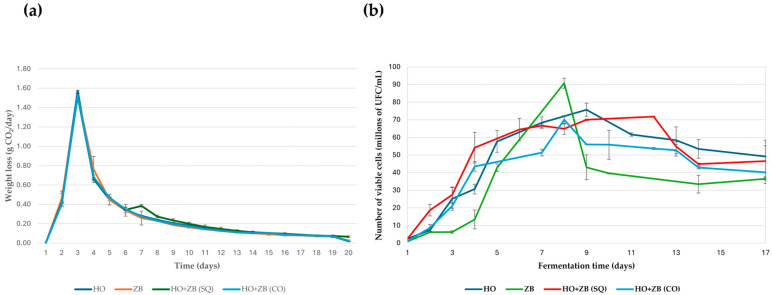
(**a**) Kinetic profiles of different fermentations performed with *Hanseniaspora opuntiae* (HO) and *Zygosaccharomyces bailii* (ZB) in monoculture. In sequential (SQ), where *H. opuntiae* was added first, and on the fourth day, *Z. bailii* was introduced and co-cultured (CO). (**b**) Viable cell count during the different fermentation conditions performed with *Hanseniaspora opuntiae* (HO) and *Zygosaccharomyces bailii* (ZB), as described in the previous figure. The means and standard deviations are represented.

Figure 3a shows the kinetic profiles obtained during the daily monitoring of each of the fermentations carried out with the yeasts *H. opuntiae* in monoculture (HO) and *Z. bailii* in monoculture (ZB), as was carried out in the study by Ansanay-Galeote et al. [35] with *Saccharomyces cerevisiae*. This graph also shows the kinetics of the co-inoculation (CO) and sequential (SQ) fermentations. These presented similar behavior to monoculture fermentations. It is noteworthy that the maximum CO_2_ release was obtained approximately 24 h after the start of fermentation in both cases. From that moment on, the fermentation rate gradually decreased until it was practically zero, marking the end of the fermentation process for the yeasts used in the trial. In the case of the sequential fermentation, a small change in the kinetic profile was observed on the fourth day due to the inoculation of *Z. bailii*. In addition to daily monitoring of the kinetic profiles of the different fermentations, the population growth of the different yeast strains was monitored daily during fermentation (Figure 3b). This study of population growth allows us to determine whether there is a relationship between fermentation kinetics and the yeast population present in the medium. In monoculture fermentations inoculated with one of the two yeast strains tested, the *Z. bailii* strain showed a significantly slower growth and survival rate than the *H. opuntiae* strain as fermentation progressed, according to the daily total cell count. Similarly, *H. opuntiae* exhibited higher viability than *Z. bailii*. On the other hand, in the mixed fermentations, the evolution of the yeast population, expressed in terms of the number of live cells, was very similar in both cases, as was the case in the individual fermentation with *H. opuntiae*. However, in the sequential fermentation, maximum viability lasted for a longer period rather than showing a prominent peak on a specific day, compared to the other fermentations performed with the *H. opuntiae* and *Z. bailii* strains. These results seem very interesting, since they suggest that a sequential fermentation, in which the *H. opuntiae* strain is inoculated first and then the *Z. bailii* strain, could be considered a strategy to complete alcoholic fermentation in the absence of *Saccharomyces cerevisiae*.

### 3.4. Oenological Parameters

In Table 1, the values shown represent averages of triplicate samples (data are mean ± standard deviation (SD)). 

The two non-*Saccharomyces* yeast species tested showed similar ethanol contents (≈10–11% *v*/*v*) in both single and mixed fermentations. However, the residual sugar concentration was lower in the mixed fermentations, although it never exceeded 5 g/L, which is characteristic of dry wines.

The fact that both yeasts are capable of consuming virtually all the sugars initially present in the culture medium (200 g/L), together with density values below the reference density of water, indicates that both non-*Saccharomyces* yeast strains are capable of complete alcoholic fermentation, achieving similar and even higher ethanol levels compared to various strains belonging to the *Saccharomyces* genus [36]. The study conducted by Del Fresno et al. [37] on the use of different yeasts for the production of rosé wines describes a strain of *Hanseniaspora vineae* capable of achieving, in a single fermentation, an ethanol content very similar to another strain of *Saccharomyces cerevisiae* (≈13% *v*/*v*) and, in addition, it achieves better results in terms of a more attractive color and the organoleptic characteristics of the final product.

Wine quality depends on multiple factors, and one of the most relevant aspects is volatile acidity, whose legal limit is 1.2 g/L for most wines. However, it is advisable to keep this as low as possible because acetic acid can be sensorially detected by the consumer at concentrations above 0.8 g/L and impart an undesirable vinegary flavor and aroma to the final product. Thus, it could be said that, in a winemaking process, it would be ideal to carry out a single fermentation with *Z. bailii*, mainly due to the fact that the volatile acidity value is below the sensory threshold, contrary to what is observed with *H. opuntiae* or in mixed fermentations with both yeasts, and the remaining oenological parameters do not differ much in either case.

### 3.5. Major Aroma and Statistical Analysis

The determined alcohol, except propanol, which has α-ketobutyric acid as its precursor, is derived from the degradation of sugars or amino acids through the decarboxylation of keto acids. These products, except 2-phenylethanol, which possesses a floral aroma, usually have solvent aromas and a higher perception threshold. Their overall concentration must not exceed 400–500 mg/L. Below, these amounts contribute to the aroma complexity of the wine [38].

As can be seen in Table 2, isoamyl alcohols show significant differences under the different conditions assayed. The highest values were observed with *H. opuntiae* and in sequential culture, whereas the lowest was reached with *Z. bailii*. The same behavior is observed with isobutanol, and the opposite is true in the case of propanol. 2-Phenylethanol provides sweet and floral aromas, as observed in the study by Gómez-Míguez et al. [39]. In our study, it was observed to exceed the perception threshold (10 mg/L) in all conditions. The lowest values were found in co-inoculation, and the highest were with *H. opuntiae* and in the sequential assay. The results obtained by Martin et al. [40] show that the concentration of 2-phenylethanol increased more in co-culture, intensifying the floral and sweet attributes of the wine.

Among the carbonyl compounds, acetaldehyde is formed by yeasts as an intermediary metabolite in the conversion of glucose into ethanol. Acetaldehyde has a pleasant, fruity aroma at minimal concentrations; however, at high levels, its odor becomes irritating and penetrating [41,42]. Usually, its concentration does not exceed 50 to 70 mg/L [38]. In this study, the amount was higher in all the cases. However, it was observed that the compounds 2,3-butanediol (meso and levo) and glycerol were produced at lower levels in the presence of *Z. bailii*. This may be due to the lower activity of glyceropyruvic fermentation. On the other hand, acetoin contributes to the development of lactic odors, such as butter. However, it only exceeds the threshold in monocultures; therefore, its use in axenic cultures would have this unique characteristic. However, in mixed cultures, a significant reduction in this compound was observed. This result is supported by the study by Capece et al. [43]. Elevated levels of acetaldehyde and acetoin are likely due to the shift in their metabolism toward glyceropyruvic fermentation to regenerate the NAD^+^ that could not be obtained through alcoholic fermentation. This would result in a balance in redox potential. At these concentrations, acetaldehyde can contribute to pungent and oxidized apple aromas. The elevated acetaldehyde levels in the different cultures are probably the result of high pyruvate decarboxylase activity in these non-*Saccharomyces* strains. Acetoin exceeded its perception threshold only in monoculture conditions (36–44 mg/L), contributing buttery notes, whereas mixed cultures showed significantly reduced levels (25–28 mg/L) below sensory relevance. This reduction in mixed cultures represents a potential advantage for the use of combined inoculation strategies.

From the sugars (glucose and fructose) present in the synthetic medium, two different pathways can be carried out. The first, through which glycerol precursors, dihydroxyacetone phosphate (dihydroxyacetone-P), are formed. This is converted into glycerol-3-phosphate (G3P) through the reduction of a molecule of NADH, through the action of the enzyme triose phosphate isomerase. Once this compound is obtained, it is converted into glycerol through the enzymatic action of glycerol-1-phosphatase, which can hydrolyze the phosphate group of G3P, forming glycerol. On the other hand, glyceraldehyde-3-phosphate is formed from glucose. There is a balance between the formation of dihydroxyacetone-P and glyceraldehyde-3-phosphate; therefore, the yeast regulates the pathway it uses to maintain this balance. From glyceraldehyde-3-phosphate, pyruvate is formed through glycolysis, producing one molecule of NADH. From pyruvate, two different pathways can occur simultaneously: First, pyruvate is transformed into acetaldehyde through decarboxylation thanks to pyruvate decarboxylase. Finally, acetaldehyde is reduced by the consumption of NADH and the action of alcohol dehydrogenase to form ethanol. The second option in the pyruvate production pathway involves the condensation of two pyruvate molecules to form α-ketolactate, catalyzed by acetolactate synthase. α-Ketolactate can be converted into several compounds, including the amino acid valine, by the action of acetolactate synthase and diacetyl, and by the action of alpha-acetolactate decarboxylase, thus preventing the formation of acetoin (a compound that imparts an undesirable flavor). When diacetyl is reduced by diacetyl reductase, acetoin (3-hydroxybutanone) is formed, which is subsequently transformed into 2,3-butanediol through a reduction reaction catalyzed by the action of the enzyme butanediol dehydrogenase [44].

This fermentation produces lactic acid, which reacts with the ethanol produced during alcoholic fermentation, where an esterification reaction occurs, forming ethyl lactate. This is also due to the acidity of the medium. As observed in Table 2, the highest values of this compound are found in the monoculture of *H. opuntiae* and in the co-culture. It is also observed that, in the presence of *Z. bailii,* the values of this ester tend to decrease, as observed in the sequential culture or monoculture of this strain, which presents the lowest values in all conditions. On the other hand, yeasts are capable of producing acetic acid as a byproduct of their metabolism, which, as mentioned above, reacts with the ethanol and triggers another esterification reaction, forming ethyl acetate *Z. bailii*, which is an osmotolerant and highly fermentative yeast; little is known about its metabolism under culture conditions or its ability to produce esters. Ethyl esters are very interesting because they have a pleasant fruity and floral aromatic note [45]. In the study conducted by Garavaglia et al. [16], it was shown that the *Z. bailii* BCV 08 strain was able to increase ester production. However, in our study, the strain used did not increase the production of major esters; rather, in the presence of this yeast, its production decreased. A greater production of this compound was observed in the monoculture of *H. opuntiae*, this is because apiculated yeasts are good producers of acetate esters, as observed in the work of Rojas et al. [45] and Filippousi et al. [46]. It was concluded that this compound decreased in the presence of *Z. bailii*, both in co-culture and sequentially. This compound provides fruity aromas, such as pineapple.

**Figure 4:**
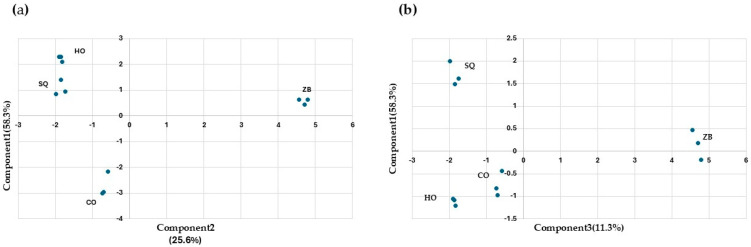
Principal component analysis of the “wines” obtained after fermentations under different conditions: *Hanseniaspora opuntiae* (HO) and *Zygosaccharomyces bailii* (ZB) in monoculture. In sequential (SQ), where *H. opuntiae* was added first, and on the fourth day, *Z. bailii* was introduced and co-cultured (CO). The analysis was performed on the major volatile compounds. (**a**) Principal component analysis between components one and two. (**b**) Principal component analysis between components one and three.

With the aim of reducing the number of variables, a principal component analysis was carried out (Figure 4). Components are a linear combination of the input variables, and the interpretation of the components obtained must be performed by the analyst. Here, the aroma compounds were selected as input variables. The first three components explain 83.4% of the observed variability (Figure 4a,b). Component 1 differentiates the ZB assay from the rest. The compounds with the greatest influence are propanol, isobutanol, and ethyl acetate. Component 2 differentiates co-inoculation from SQ and HO and is mainly influenced by 2,3-butanediol (levo), 2-phenylethanol, and diethyl succinate. Lastly, component 3 differentiates SQ from HO, and the most influential compounds are 2,3-butanediol (meso), acetaldehyde, and ethyl lactate.

## 4. Conclusions

This study demonstrates that non-*Saccharomyces* yeasts, specifically *Hanseniaspora opuntiae* and *Zygosaccharomyces bailii*, isolated from challenging oenological environments, possess significant potential to improve wine quality through their dual *β*-glucosidase activity and killer phenotype. These traits are crucial for the release of aroma-enhancing compounds and for providing a natural form of microbial competition during fermentation, positioning these strains as valuable alternatives to traditional fermentative yeasts. Our results show that *H. opuntiae* is particularly effective when applied during the early stages of fermentation, where its *β*-glucosidase activity enhances the release of fruity and floral aromas, contributing to a more complex and appealing sensory profile. In contrast, *Z. bailii* excels in completing fermentation, even under high-sugar conditions, and is associated with lower volatile acidity production, which is essential for maintaining wine freshness and avoiding undesirable vinegary notes. Sequential inoculation—introducing *H. opuntiae* first, followed by *Z. bailii*—proved especially beneficial. This strategy achieved complete fermentation, reduced residual sugar, and resulted in wines with enhanced aromatic complexity and improved mouthfeel, even in the absence of *Saccharomyces cerevisiae*. These findings provide practical guidance for winemakers: employing *H. opuntiae* at the start of fermentation and introducing *Z. bailii* after several days can optimize both fermentation kinetics and sensory outcomes. Fermentation temperatures around 21 °C and careful timing of inoculation are recommended to maximize the performance of these strains. Importantly, this work challenges the traditional view of these non-*Saccharomyces* species as spoilage organisms. Instead, it highlights their suitability as bio-tools for precision oenology, capable of enhancing varietal expression and contributing to the development of wines with distinctive sensory attributes. The application of these strains in commercial winemaking could offer new avenues for aroma enhancement, microbial control, and adaptation to musts with high sugar or ethanol content.

Future studies will focus on validating these findings in real grape must fermentations and further exploring the sensory and technological impacts of these yeasts under commercial winemaking conditions.

## Figures and Tables

**Table 1:** Oenological parameters.

Oenological Parameters	*H. opuntiae*	*Z. bailii*	Co-Inoculation	Sequential
Volatile acidity (g/L)	1.05 ± 0.00 ^b^	0.64 ± 0.06 ^a^	1.02 ± 0.00 ^b^	1.03 ± 0.06 ^b^
Reducing sugar(g/L)	3.92 ± 0.03 ^a^	4.11 ± 0.07 ^a^	0.83 ± 0.00 ^a^	0.70 ± 0.09 ^a^
Ethanol (%, *v*/*v*)	10.2 ± 0.1 ^a^	10.2 ± 0.1 ^a^	10.9 ± 0.1 ^c^	10.7 ± 0.2 ^b^

Values with different superscript letters in the same row are significantly different test (*p* < 0.05).

**Table 2:** Major aroma compounds. The values shown represent averages of triplicate samples (data are mean ± SD). Values with different superscript letters in the same row are significantly different test (*p* < 0.05).

Compounds (mg/L)	*H. opuntiae*	*Z. bailii*	Co-Inoculation	Sequential (SQ)
Alcohols				
1-Propanol	4.0 ± 0.3 ^a^	28 ± 1 ^b^	4.5 ± 0.4 ^a^	4.7 ± 0.3 ^a^
Isobutanol	72 ± 5 ^c^	20 ± 1 ^a^	66 ± 2 ^b^	73 ± 3 ^c^
Isoamyl alcohols	205 ± 13 ^bc^	130 ± 7 ^a^	189 ± 6 ^b^	211 ± 10 ^c^
2-Phenylethanol	40 ± 2 ^c^	35 ± 1 ^b^	27 ± 2 ^a^	36 ± 3 ^bc^
Carbonyl Compounds				
Acetaldehyde	129 ± 14 ^a^	154 ± 7 ^b^	158 ± 12 ^b^	174 ± 18 ^b^
Acetoin	36 ± 3 ^b^	44 ± 3 ^c^	25 ± 2 ^a^	28 ± 2 ^a^
Esters				
Ethyl lactate	22 ± 2 ^b^	13.4 ± 0.9 ^a^	24 ± 2 ^b^	16 ± 2 ^b^
Diethyl succinate	19 ± 2 ^b^	18 ± 1 ^b^	7 ± 1 ^a^	9.30 ± 0.81 ^a^
Ethyl acetate	31 ± 3 ^c^	5.1 ± 0.2 ^a^	22 ± 3 ^b^	22 ± 3 ^b^
Polyols				
2,3-Butanediol (levo)	286 ± 15 ^b^	187 ± 8 ^a^	208 ± 3 ^a^	272 ± 19 ^b^
2,3-Butanediol (meso)	92 ± 6 ^b^	113 ± 8 ^c^	75 ± 2 ^a^	90 ± 8 ^b^
Glycerol (g/L)	4.60 ± 0.10 ^b^	2.6 ± 0.3 ^a^	2.80 ± 0.04 ^a^	4.6 ± 0.4 ^b^

## Data Availability

The original contributions presented in this study are included in the article. Further inquiries can be directed to the corresponding author.
